# Using critical consciousness to inform health professions education: A literature review

**DOI:** 10.1007/s40037-016-0324-y

**Published:** 2017-01-03

**Authors:** Mark Halman, Lindsay Baker, Stella Ng

**Affiliations:** 1 Department of Psychiatry, Mount Sinai Hospital, University of Toronto, Toronto, ON Canada; 2 St. Michael’s Hospital, University of Toronto, Toronto, ON Canada

**Keywords:** Critical consciousness, Critical pedagogy, Health professions education, Literature review

## Abstract

**Purpose:**

To explore how, in health professions education (HPE), the concept of critical consciousness has been defined and discussed, and to consider and suggest how critical pedagogy could be applied in practice. This exploration responds to increasing calls in the literature for HPE to foster compassionate care and social consciousness through the social sciences and humanities.

**Method:**

The authors searched Medline/PubMed, ERIC and Web of Science for articles focusing on critical consciousness and/or critical pedagogy involving health professions. A thematic analysis aimed to identify key themes of critical consciousness in HPE literature.

**Results:**

The authors included 30 papers in their review. Key themes related to defining and discussing core attributes of critical consciousness in HPE were: 1) appreciating context in education and practice; 2) illuminating power structures; 3) moving beyond ‘procedural’; 4) enacting reflection; and 5) promoting equity and social justice.

**Conclusions:**

Critical consciousness may inform an appropriate critical pedagogy for fostering compassionate, humanistic, socially conscious health professionals who act as agents of change. While the authors share critical teaching practices for educators, considerable care must be taken in efforts to use critical pedagogy within the current structures of HPE programmes. The authors suggest attending to the philosophical and theoretical origins of critical consciousness and those of the dominant models of contemporary HPE (e. g. competency-based approaches) in order to ensure the tenets of critical pedagogy can be enacted authentically.

## What this paper adds

Critical consciousness, an education concept originating from emancipatory work with marginalized and disadvantaged populations, holds promise if health professions education (HPE) aims to foster compassionate and socially responsible providers. But a gap remains in understanding how such a critical approach can be practically applied, particularly in the face of dominant competency-based curricula. We conducted a literature review to summarize and present ways in which critical consciousness has been explored in HPE. We share five core themes to advance dialogue about critical consciousness in HPE, as well as practical strategies for educators to consider and enact.

## Introduction

Fostering compassionate, socially responsible health professionals is an imperative of health professions education (HPE). A number of papers in HPE have argued that competency-based curricula fall short of this imperative [1–5]. Competency-based medical education runs the risk of reducing learning to a series of measurable skills and behaviours. Critically, this narrow focus potentially obscures professional identity development [6] and the place of personhood and values in becoming a physician [7].

Critical approaches to education are often recommended in response to these concerns [1–5]. Critical approaches explore unexamined assumptions that are held at individual, institutional, and cultural levels of healthcare and aim to raise awareness of the conditions of the people and communities served. They question the power relations inherent in health and healthcare, and how individuals, groups, and systems may be (unintentionally or intentionally) complicit in perpetuating and reproducing the current, at times inequitable, state of social conditions [8, 9]. Yet a gap remains: what would critical approaches look like in actual HPE practice and how could they be applied within the current structures of HPE?

### Critical consciousness

*Critical consciousness*, one such critical approach, may help to fill this gap. The concept of critical consciousness was advanced by educationalist Paulo Freire in the 1970s, while he worked to transform education amongst the poor and illiterate in Brazil [10]. To summarize, Freire’s theory of critical consciousness critiqued the banking model of education, instead proposing transformative education through dialogue, and required a reflective awareness of and action upon societal conditions and inequities. Each of these concepts will now be explained in turn.

Freire criticized the traditional banking model of education, wherein the teacher deposits knowledge into the learner, the recipient of knowledge. Freire argued that the banking model reproduced knowledge that maintained the status quo, and thus maintained the oppression of already marginalized populations. Freire asserted that this type of learning was shaped and constrained by power relations, and the associated inequities of such relations embedded in social structures. Therefore, in order to foster *transformative* knowledge that would be liberating instead of oppressing, he argued that learners needed to connect with their own personal, cognitive, and emotional experience, to engage with others through dialogue (engaged discussion that includes affective and experiential knowledge) and to emancipate themselves and others through praxis (the realization of theory within action). This transformative approach to education, as opposed to the banking model, would challenge dominant, long-held beliefs, to truly transform society. This capacity to connect with one’s situated position in society and to engage in dialogue about inequities rested upon the concept of conscientization. Conscientization meant a reflective reading of the world, with focused attention upon societally embedded inequities. From this conscientization, learners would be implored to act as agents of change [10]. ‘Conscientization is both cognitive and affective, and leads to engaged discourse, collaborative problem-solving, and a ‘rehumanization’ of human relationships’ [1, p. 783].

### Critical consciousness in HPE

While other movements (e. g. narrative medicine, shared decision-making, and person-centred care) share some common ground with critical consciousness, we argue that critical consciousness and its related critical pedagogy remain under-examined and uniquely valuable in HPE. By critical pedagogy, we mean the philosophy of education and practice of teaching informed by critical consciousness. In a time of increasing advocacy for the disadvantaged and globalization of HPE [11], we suggest Freire’s emancipatory approach is particularly useful for HPE, even while the other related approaches may also be helpful. A critical stance allows one to notice the social and political nature of education and healthcare, the influences of power and privilege in the delivery of care, and the ways in which all learners as individuals and as members of a healthcare culture can contend with unexamined assumptions that foster oppression. Therefore, a critical pedagogy may be necessary if we are to ensure personhood and critical consciousness develop alongside competencies and that HPE systems do not inadvertently create a cadre of providers skilled in reproducing desired competencies but unable to advance the increasing social responsibility agenda of healthcare.

The current climate of HPE, with its renewed focus on compassionate care, advocacy and equity, and globalization, combined with the call for critical and humanistic approaches to support this focus, justifies our review paper. Although critical consciousness is not per se ‘new’, it remains understudied within the HPE context. So, in order to advance a more complete and authentic understanding of and dialogue about critical consciousness in the context of HPE, we conducted a review of the literature. We aimed to: 1) explore how critical consciousness is defined and discussed in HPE literature and 2) informed by this literature review, discuss how critical pedagogy could be applied in HPE practice. By synthesizing the literature, we hope that critical consciousness, and its associated critical pedagogy, can begin to be considered thoughtfully in response to the repeated calls to foster compassionate and socially conscious health professionals. By extending possible critical pedagogical practices, we hope this paper provides support to critically oriented educators.

## Methods

To explore current manifestations of critical consciousness in HPE we conducted a review of the literature. Three databases were searched by a health sciences librarian and the authors using a combination of keyword terms and database-specific subject headings (critical consciousness, critical pedagogy, curriculum, education, training, medical, health professions, nursing, clinical teaching): Medline/PubMed (1946 to 14 May 2014), ERIC on the ProQuest Platform (1966 to 14 May 2014), and Web of Science (1900 to 14 May 2014). Searches were limited to peer reviewed, English-language articles. We included articles focusing on critical consciousness and/or critical pedagogy involving at least one core health profession, which we defined as well-established or regulated health professionals (e. g. medicine, nursing, or health disciplines). Therefore, articles focusing on unregulated or informal health workers (e. g. community/family members) were excluded in order to maintain a focus on HPE.

**Fig. 1:**
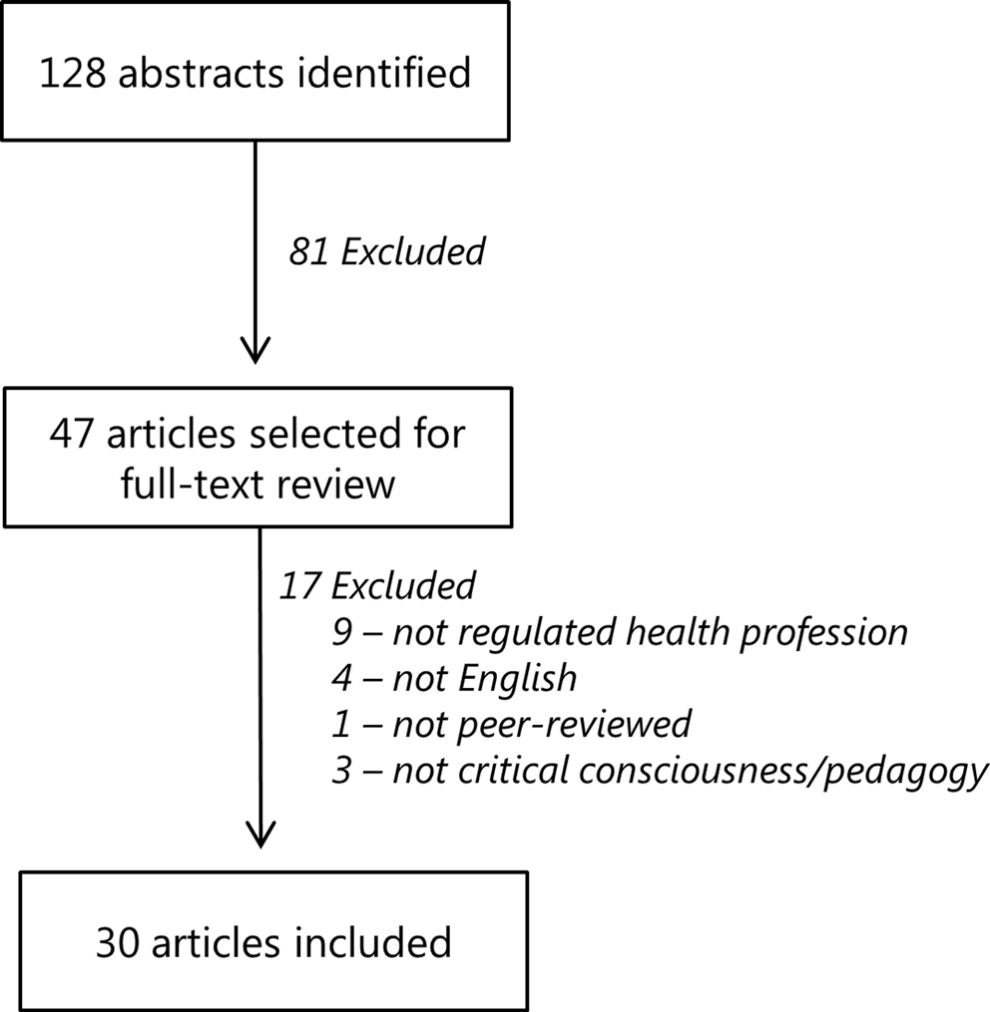
Flowchart of literature search and article selection process from a critical review of the literature on critical consciousness/critical pedagogy published before May 2014

After duplicates were removed by the librarian, a total of 128 abstracts were identified and shared with the review team (MH, LB, SN). Titles and abstracts were then reviewed for relevance by the team and a total of 47 articles were selected for full text review (Fig. 1). The criteria for relevance at this stage again focused on selecting articles about critical consciousness and/or critical pedagogy involving at least one core health profession, which we again defined as well-established or regulated health professionals (e. g. medicine, nursing, or health disciplines).

Two members of the team (MH and SN) read and re-read each full text article and abstracted pertinent data onto a data extraction sheet. This data extraction sheet was developed after piloting with a subset of articles and meeting and discussion by the team. The final extraction sheets captured demographic information (author profession, country, journal, year of publication), article type (e. g. review, empirical, conceptual, descriptive), definitions of critical consciousness, article themes, and specific practices of critical pedagogy.

Article themes were conceptualized/abstracted as core ways in which article authors represented the concepts of critical consciousness in their work. Importantly, the research team based these themes on their own interpretations of what the article authors presented. Thematic abstraction was conducted with each article serving as an analytic unit, and using an interpretative qualitative lens such that the main messages of the article were documented. Through an iterative process, with regular discussion amongst team members, redundant themes were collapsed into representative theme headings. A third member of the team (LB) was consulted if a difference of opinion arose. During the data extraction phase an additional 17 articles were excluded because they did not meet criteria for inclusion following detailed full-text review, resulting in 30 articles included in this review.

During the abstraction process, the team also attended to critical pedagogy practices worth discussing. Therefore, particularly relevant (to the HPE goals of fostering compassionate and socially conscious practitioners) practices of critical pedagogy, as discussed in the articles and noted during abstraction, are shared in the discussion and implications section of this article.

## Results

Of the 30 articles included in the review, the majority were conceptual (theoretical or perspective pieces) (*n* = 13) [1, 12–23], though a number also described (*n* = 8) [1, 19, 21, 24–28] and/or evaluated (*n* = 8) [26, 27, 29–33], educational programmes, reported on empirical research (*n* = 7) [18, 29, 34–38] or conducted reviews of the literature (*n* = 2) [25, 39]. Almost half of the articles were published within the last five years (*n* = 14), and the majority were written by authors in the nursing profession (*n* = 20). While most authors came from the United States (*n* = 10), Canada (*n* = 7), or the United Kingdom (*n* = 6), others represented New Zealand, Finland, Germany, Australia, Brazil, Pakistan and South Africa.

We identified five *overlapping* themes relating to defining and discussing core attributes of critical consciousness in HPE: (1) Appreciating context in education and practice; (2) Illuminating power structures; (3) Moving beyond procedural; (4) Enacting reflection and (5) Promoting equity and social justice. Note that while in Table 1 we have represented the themes covered within each article we reviewed, these selections represent only the predominant foci of each article as interpreted by this paper’s research team. Below, we define and articulate each theme individually, using exemplary quotations from the articles to illustrate each.

**Table 1:** Articles included in our review

First author	Year	Journal	Predominant Themes in articles				
			Appreciating context	Illuminating power structures	Moving beyond procedural	Enacting reflection	Promoting equity and social justice
Andre [32]	1999	Contemporary Nurse		x			
Bowman [12]	1995	Nurse Education Today	x				x
Chiesa [26]	2007	International Nursing Review	x				x
Clare [13]	1993	Nurse Education Today		x		x	
Donetto [18]	2012	British Journal of Sociology of Education			x		
Fleming [14]	2007	Health Education Research				x	
Getzlaf [24]	2010	International Journal of Nursing Education Scholarship				x	
Grace [15]	2013	Advances in Nursing Science			x		
Hanson [41]	2011	Journal of Studies in International Education			x		x
Harden [16]	1996	Nurse Education Today		x			
Hartrick [19]	1998	Journal of Nursing Education		x	x		
Hawks [20]	1992	Journal of Advanced Nursing		x		x	
Hedin [29]	1987	International Journal of Nursing Studies		x	x		
Hezekiah [21]	1993	Journal of Nursing Education		x		x	
Ironside [39]	2001	Advances in Nursing Science			x		x
Kumagai [30]	2007	Medical Teacher			x		x
Kumagai [1]	2009	Academic Medicine			x		x
Liimatainen [34]	2001	Journal of Advanced Nursing				x	
Mabhala [37]	2013	International Journal for Equity in Health	x				x
McDowell [37]	2012	Journal of Marital and Family Therapy	x			x	
Mikol [40]	2005	Nursing Education Perspectives	x		x		
Milligan [22]	1995	Nurse Education Today			x		
Perron [23]	2010	Advances in Nursing Science		x			
Pitner [17]	2005	American Journal of Orthopsychiatry	x	x			
Platt [27]	2012	Journal of Marital and Family Therapy	x				
Racine [28]	2012	Journal of Transcultural Nursing					x
Reid [33]	2011	Education for Health		x			x
Ross [31]	2011	Journal of Continuing Education in the Health Professions				x	x
Schiff [25]	2012	Hawaii Journal of Medicine and Public Health					x
Sharples [38]	2013	Nursing Education Today		x			

### Appreciating context in education and practice

Understanding and appreciating the personal contexts of individual learners, and the contexts within which learning and practice takes place, is a central focus [12, 17, 26, 27, 35, 37, 40]. Articles highlight how personal history and lived experiences – as individuals’ contexts – impact the educational experience and the learner’s lens for new knowledge. For example, rather than negating or ignoring the impact of personal experience on learning, Chiesa et al. argue for incorporating personal experiences in learning [26]. In their process, questions emerged from the group leading to local level strategies to overcome social exclusion in the healthcare sector. And Bowman highlights the value of examining personal context in ethics education, in order to engage students in understanding themselves, their biases, and others’ perspectives [12].

The articles also highlight the importance of appreciating context as the communities, systems and broader relationships within which learning and practice takes place. McDowell et al. [35] describe the value of *immersive* experiences in their learning programme as a way in which to foster contextual awareness to meaningfully add a critical dimension to their global health work. They talk about personal transformations that participants experience which include ‘greater recognition of one’s own privilege(s), enhanced sense of social responsibility, improved ability to think contextually and systematically … and a greater readiness to work from a liberatory therapeutic framework that supports social equity’ [35, p. 376–7]. Central to this theme is the human context within which learning and practice takes place. Maintaining this core aspect of personhood is essential to the critically conscious learner and practitioner.

### Illuminating (and changing) power structures

Articles addressed the effects of power relations on educational and healthcare processes and aimed to make explicit the exploration of how power impacts interactions in these settings [13, 16, 17, 19–21, 23, 29, 32, 33, 38]. Power relations were examined in terms of the power healthcare professionals hold in patient interactions, empowerment of communities of citizens and patients, as well as the emancipation of professional groups from oppressive structures and relations. Overall, the illumination of power structures tended to be portrayed as a theoretically and practically essential component of critical consciousness.

Articles recommended the explication of power relations with *learners*. Reid [33] argues that if we want education to be transformative for learners so that they challenge accepted norms and power structures, then we need to explicitly discuss power issues throughout their education. Unless power is made explicit and visible, medical schools will continue to reproduce graduates with the same values and perspectives as their teachers. This illumination of power structures is linked with a social justice imperative, which is crucial ‘in this era of increasing disparity in health status and access to healthcare’ [33, p. 6]. Although power dynamics are pervasive in the healthcare system, it is rare that conventional education systems promote their awareness, leading to unexamined perpetuation of system level exclusions.

Several of the papers, particularly from the nursing field, assert a need for *professionals* to seek emancipation from oppressive power relations within the healthcare system. They argue that until this happens, they will be unable to engage in true humanistic care [16]. Harden calls on nurses to attend to ‘the pursuit of radical change through the constant questioning and critiquing of unacceptable conditions in which certain people and groups in our society are forced to live’ [16, p. 36]. Similarly, Clare [13] urges nurses to challenge the taken-for-granted dominance of particular types of knowledge and values over others. She writes: ‘the first step is to fully understand that education is a political act and that knowledge is inextricably related to power.’ [13, p. 285] She calls on nurses to question – and to come together as communities to question – and critique the systems within which they work. Only through collective challenges, or advocacy, they argue, will professional action occur that transforms the status quo [13, 16].

### Moving beyond procedural

Freire’s [10] original critique of the status quo of education as a *banking model* pervades the articles we reviewed, as they resist the ‘knowledge, skills, and attitudes’ breakdown characterizing much of the HPE literature. Freire defined the ‘banking model of education’ as one in which facts are deposited into passive learners [10]. Similarly, the articles we reviewed present arguments for broader definitions of knowledge and learning than those required of a banking model [1, 15, 18, 19, 22, 29, 30, 39–41].

In an ethnographic study examining students’ understanding of patient-centred practices, Donetto [18] highlights the potential limitations of oversimplified approaches to teaching core attitudes such as empathy and their evaluation in OSCE settings through largely behaviourist approaches. She discusses how concepts that aim to encourage meaningful interaction and engagement with patients can become ‘artificial performances’, with students simply acting out empathy. She argues that the traditional behaviourist approaches reproduce forms of learning that fail to critically analyze key social and political aspects of the health professional role. She asserts that a critical pedagogy may instead foster ‘critical insight into the possible contexts, meanings and effects of medical practices amongst students [and] may contribute to the development of professionals that are better equipped to engage with the complexities and tensions of clinical interactions’ [18, p. 444].

Many echoed this assertion of how critical consciousness could help overcome the limited, instrumentally and technically focused view of our current HPE curricula, for example with respect to cultural competency. Kumagai [1] explains that ‘*cultural competency is not an abdominal exam*. It is not a static requirement to be checked off some list but is something beyond the rigid categories of knowledge, skills and attitudes: the continuous critical refinement and fostering of a type of thinking and knowing … of self, others and the world’ [1, p. 783].

### Enacting reflection

Another prominent theme in the articles reviewed is the notion of *enacting *reflection [13, 14, 20, 21, 24, 31, 34, 35]. While reflection is certainly a popular focus of medical and HPE literature (e. g. [9, p. 39–42]), in the articles reviewed there was a notable emphasis on *acting* upon reflection rather than ‘demonstrating’ or ‘performing’ reflection for reflections’ sake, or for an individual’s personal development. In the theoretical framing of critical consciousness, reflection without social action is insufficient. Instead, the articles describe critically oriented reflection; reflection through a lens of power and structure compels people to act, and to transform the status quo. In highlighting the importance of facilitating transformative action within education, Clare [13] notes that if we fail to link knowledge to the personal, social and political interests then ‘our attempts to empower students, or to produce reflective practitioners … will come to little. Students may become self-critical but they will not be socially critical – a prerequisite for developing a critical consciousness in order to transform practice’ [13, p. 285].

Yet the imperative to act based on critical reflection ironically faces the tension of individual agency versus the constraints of structure. In discussing the value of reflection to health education, Fleming [14] notes that the absence of perceived or actual agency may be a key barrier to reflection. He writes that ‘reflection may bring about the desire for change and progress which may be difficult or impossible to realize in specific organizational contexts, leading to frustration and discontent’ [14, p. 662]. Still, articles stress the importance of creating the educational space for critical reflection as a first step in creating awareness that action may even be needed. Awareness is a prerequisite to action, and may be linked with action, as it means individuals’ ways of seeing the world and acting will change and, collectively, transform health and healthcare [24].

### Promoting equity and social justice

Essential to all of the included articles is the explicit goal that healthcare providers work to promote equity and social justice in their communities and view this quest for social justice as a core element of their education and practice [1, 12, 25, 26, 28, 30, 31, 33, 37, 39, 41]. Health professionals would not be critically conscious if they practised as if health and healthcare exists in a socio-political vacuum, ignoring the influence of social determinants of health. For example, Chiesa argues that health professionals should work from a guiding perspective to ‘broaden the conditions so that the opportunities can be equal, reducing the influences of inequality resulting from a position in the social structure’ [26, p. 401]. Similarly, Ross reports on a faculty development strategy that ‘emphasizes the development of critical awareness of disparities and a commitment to address social justice’ [31, p. 192] as a way towards advancing the educational commitment to overcoming well described racial and ethnic health disparities. In another example, Mabhala finds that nurse educators were working from a critical pedagogy in order to address issues of inequity in public health. ‘These findings … led to the conclusion that social justice was the underpinning principle behind participants’ public health vision. It emerged in this study that embodying social justice requires nurses who possess a wider vision of public health – the ability to see the connectedness between public health and the wider socioeconomic and political systems that produce and sustain inequalities in health’ [37, p. 9].

## Discussion and conclusions

The themes presented in the findings section represent the ways in which critical consciousness appears in the HPE literature we reviewed. In this section we will discuss limitations, possible practices of critical pedagogy for health professions educators, and considerations for the field of HPE.

Notably, few of the papers critically examine the potential limitations of adopting critical consciousness approaches. Embodying critical consciousness requires a willingness to challenge one’s own position of power and privilege. In the health professions, both learners and teachers may experience privilege, yet a sense of disempowerment, when introduced to the concept of critical consciousness and the examination of bias, identity, and their own situated position in society [17]. HPE learners, despite being a privileged group either before or once they have entered HPE, may be at a disadvantaged and disempowered position in their educational context. They may be overwhelmed by the volume of new material to learn and uncertain of their position in the changing healthcare system. Pitner and Sakamoto [17] note that the pursuit of critical consciousness is both anxiety provoking and ‘requires a high level of cognitive resource’ [17, p. 688]. This anxiety may be disadvantageous to the learner looking to secure their position in a competitive learning environment. The taxing nature of the pursuit may paradoxically trigger emotionally distancing reactions and become a barrier to engagement rather than a strength. Similarly, clinical faculty may feel disempowered by an emancipatory approach to learning and healthcare, which may not be supported at a systems and structural level. It can be challenging for discussions of – and challenges to – power and privilege to take place in an environment where learners and faculty may already feel their positions are precarious. We suggest that tapping into this sense of discomfort may actually be a way to engage the sense of a common humanity embedded in critical consciousness.

We appreciate that educators will wonder how, specifically, to put critical consciousness into practice within HPE. Therefore, in Table 2, we fulfil the second aim of this paper: to suggest critical pedagogical practices for educators. In this table, we present specific critical pedagogical practices and actions, gleaned from the literature review, which seem feasible and meaningful in relation to HPE’s goals of fostering compassionate, socially conscious practitioners. At the same time, recognizing the complexity of critical approaches, we stress the need to avoid oversimplification or to use a ‘recipe’ approach for critical pedagogy.

**Table 2:** Common practices of a critical pedagogy

Common practice	Rationale and Description	Examples
Promote authentic dialogue	Dialogue promotes the authentic exchange of ideas. It moves beyond discussion. It begins in a safe learning space and invites learners to openly share their experiences without concern for judgment	[22, 35]
Recognize the value of everyone in the room	Faculty are not the authority on the learning experience in these situations because they are not the authority on the lived experience of the learner or the patient. The value of everyone in the room is recognized and learners are experts of their own expertise. Taking such a position creates a supportive and egalitarian atmosphere	[17, 39]
Share and invite stories	Learners can acquire personal knowledge through the narrative of people who experience health and healthcare. This knowledge differs from biomedical forms and purposes of knowledge, and matters to critical consciousness. Personal narratives are shared with learners and patients and other relevant individuals are invited to share their stories	[1, 20, 24, 37]
Question the status quo	Much of what we do in healthcare is because we have always done it that way and therefore we take it for granted – we’ve stopped ‘seeing it.’ Learners bring fresh views; if learners are empowered to ask questions, we may enable more questioning and transforming of the status quo. Ask learners why we do things the way we do them, and how our current approach may be perpetuating inequity or injustice	[40, 41]
Create cognitive disequilibrium	Cognitive disequilibrium refers to a state of cognitive imbalance. We experience such a state when encountering information that requires us to develop new schema or accommodate existing schema. Facilitate encounters with the unfamiliar for learners in order to stimulate the examination of their values and beliefs	[1, 31]
Challenge the power hierarchy	Power dynamics are inherent in health professions education and influence what is safe, and what is possible for learners to say and do. Acknowledge this power hierarchy and actively challenge it	[1, 23, 29, 39]

While these common practices may be helpful to critically oriented educators, moving critical pedagogy forward in HPE in a comprehensive way will require attention to the underlying philosophical and theoretical origins of both critical and competency-based pedagogies. As others have noted, the dominant epistemological positions of HPE can distort educational approaches, borrowed from other fields and thus deriving from differing epistemological bases, when these approaches are adopted into HPE [9]. For example, in the current climate, an attempt to add a critical consciousness orientation to an entire curriculum should be mindful of the current predominance of competency-based medical education, and *its* philosophical and theoretical origins.

We would be remiss not to briefly discuss other approaches to foster compassionate, socially conscious, person-centred health professionals. For example, narrative medicine aims to restore and honour persons’ stories to clinical practice, research, and education. This restoration claims to encourage reflection, promote empathy, and centre care around people as a whole [42]. Person-centred care aims for a relational and systems-based approach to practice [43]. Shared decision-making aims to restore choice to individuals with regard to their own care, and has an ethical imperative as its basis [44]. One might wonder, then, what Freire’s critical pedagogy really offers beyond these other approaches. We argue that a critical pedagogy could certainly co-exist with these approaches. Yet an examination of each, their historical and philosophical roots, and their contextualized outcomes and associated mechanisms of change would be needed in order to integrate them effectively. Further, we argue that of all of these approaches, Freire’s critical pedagogy most centrally focuses upon emancipation of the marginalized and disadvantaged. Therefore, at a time when HPE is seeing a rise in advocacy and globalization, Freire’s theories are welcome and needed additions to HPE’s repertoire of theories and approaches [45, 46]. This article is an early step; more (empirical and theoretical) work is needed to update and contextualize Freire’s theories for our current HPE contexts.

## Summary

Existing approaches to HPE, such as competency-based medical education that strives to guide health professions learners towards desirable attitudes and behaviours may, when applied uncritically, perpetuate existing problems. Critical consciousness, with its emancipatory history, holds the potential to transform rather than reproduce current problems. The desired outcome of a critical pedagogy is an authentically and critically attuned carer who fundamentally embodies core human values of social justice within a commitment to continually improve upon the status quo. This paper summarizes current understandings and applications of critical pedagogy in HPE. Attempting to apply critical pedagogy, however, requires nuanced and ongoing attention to how it fits and conflicts with prevailing approaches in HPE.

## References

[1] Kumagai A, Lypson ML (2009). Beyond cultural competence: critical consciousness, social justice, and multicultural education. Acad Med.

[2] Kumagai AK (2012). Perspective: acts of interpretation. A philosophical approach to using creative arts in medical education. Acad Med.

[3] Betancourt JR (2003). Cross-cultural medical education: conceptual approaches and frameworks for evaluation. Acad Med.

[4] Wear D (2003). Insurgent multiculturalism: rethinking how and why we teach culture in medical education. Acad Med.

[5] Martimianakis MAT, Michalec B, Lam J, Cartmill C, Taylor JS, Hafferty FW (2015). Humanism, the hidden curriculum, and educational reform: a scoping review and thematic analysis. Acad Med.

[6] Jarvis-Selinger S, Pratt DD, Regehr G (2012). Competency is not enough: integrating identity formation into the medical education discourse. Acad Med.

[7] Whitehead C, Selleger V, van de Kreeke J, Hodges B (2014). The ‘missing person’ in roles-based competency models: a historical, cross-national, contrastive case study. Med Educ.

[8] Hodges B (2014). When I say … critical theory. Med Educ.

[9] Ng SL, Kinsella E, Friesen F, Hodges B (2015). Reclaiming a theoretical orientation to reflection in medical education research: a critical narrative review. Med Educ.

[10] Freire P (1993). Pedagogy of the oppressed: 20th anniversary edition.

[11] Law M, Leung P, Veinot P, Miller D, Mylopoulos M (2016). A qualitative study of the experiences and factors that led physicians to be lifelong health advocates. Acad Med.

[12] Bowman A (1995). Teaching ethics: telling stories. Nurse Educ Today.

[13] Clare J (1993). Change the curriculum – or transform the conditions of practice?. Nurse Educ Today.

[14] Fleming P (2007). Reflection – a neglected art in health promotion. Health Educ Res.

[15] Grace PJ, Perry DJ (2013). Philosophical inquiry and the goals of nursing. Adv Nurs Sci.

[16] Harden J (1996). Enlightenment, empowerment and emancipation: the case for critical pedagogy in nurse education. Nurse Educ Today.

[17] Pitner RO, Sakamoto I (2005). The role of critical consciousness in multicultural practice: examining how its strength becomes its limitation. Am J Orthopsychiatry.

[18] Donetto S (2012). Medical students and patient-centred clinical practice: the case for more critical work in medical schools. Br J Sociol Educ.

[19] Hartrick G (1998). A critical pedagogy for family nursing. J Nurs Educ.

[20] Hawks JH (1992). Empowerment in nursing education : concept analysis and application to philosophy, leaming and instruction. J Adv Nurs.

[21] Hezekiah J (1993). Feminist pedagogy: a framework for nursing education?. J Nurs Educ.

[22] Milligan F (1995). In defence of andragogy. Nurse Educ Today.

[23] Perron A, Rudge T, Blais A-M, Holmes D (2010). The politics of nursing knowledge and education of the militarization of nursing. Adv Nurs Sci.

[24] Getzlaf B, Osborne M (2010). A journey of critical consciousness: an educational strategy for health care leaders. Int J Nurs Educ Scholarsh.

[25] Schiff T, Rieth K (2012). Projects in medical education: ‘Social Justice in Medicine’ a rationale for an elective program as part of the medical education curriculum at John A. Burns School of Medicine. Hawaii J Med Public Health.

[26] Chiesa M, Fracolli L (2007). An educational process to strengthen primary care nursing practices in São Paulo, Brazil. Int Nurs Rev.

[27] Platt JJ (2012). A Mexico City-based immersion education program: training mental health clinicians for practice with Latino communities. J Marital Fam Ther.

[28] Racine L, Proctor P, Jewell LM (2012). Putting the world as classroom: an application of the inequalities imagination model in nursing and health education. J Transcult Nurs.

[29] Hedin B (1987). Nursing education and social constraints: an indepth analysis. Int J Nurs Stud.

[30] Kumagai AK, White CB, Ross PT, Purkiss J, O’Neal CM, Steiger J (2007). Use of interactive theater for faculty development in multicultural medical education. Med Teach.

[31] Ross PT, Kumagi AK, Joiner TA, Lypson ML (2011). Using film in multicultural and social justice faculty development: scenes from crash. J Contin Educ Health Prof.

[32] Andre C, Hall C (1999). Nurses re-entering the workforce: a special needs group. Contemp Nurse.

[33] Reid SJ (2011). Pedagogy for rural health. Educ Health (Abingdon).

[34] Liimatainen L, Poskiparta M, Karhila P, Sjögren A (2001). The development of reflective learning in the context of health counselling and health promotion during nurse education. J Adv Nurs.

[35] McDowell T, Goessling K, Melendez T (2012). Transformative learning through international immersion: building multicultural competence in family therapy and counseling. J Marital Fam Ther.

[36] Harris DL, Krause KC, Parish DC, Smith MU (2007). Academic competencies for medical faculty. Fam Med.

[37] Mabhala M (2013). Health inequalities as a foundation for embodying knowledge within public health teaching: a qualitative study. Int J Equity Health.

[38] Sharples N (2013). An exploration of deaf women’s access to mental health nurse education in the United Kingdom. Nurse Educ Today.

[39] Ironside PM (2001). Creating a research base for nursing education: an interpretive review of conventional, critical, feminist, postmodern, and phenomenologic pedagogies. Adv Nurs Sci.

[40] Mikol C (2005). Teaching nursing without lecturing: critical pedagogy as communicative dialogue. Nurs Educ Perspect.

[41] Hanson L, Harms S, Plamondon K (2011). Undergraduate international medical electives: some ethical and pedagogical considerations. J Stud Int Educ.

[42] Greenhalgh T, Hurwitz B (1999). Narrative based medicine: why study narrative?. BMJ.

[43] Starfield B (2011). Is patient-centered care the same as person-focused care?. Perm J.

[44] Elwyn G, Frosch D, Thomson R (2012). Shared decision making: a model for clinical practice. J Gen Intern Med.

[45] Toumi R (2014). Globalization and health care: global justice and the role of physicians. Med Health Care Philos.

[46] Martimianakis MA, Hafferty FW (2013). The world as the new local clinic: a critical analysis of three discourses of global medical competency. Soc Sci Med.

